# Photogrammetric Process to Monitor Stress Fields Inside Structural Systems

**DOI:** 10.3390/s21124023

**Published:** 2021-06-10

**Authors:** Leonardo M. Honório, Milena F. Pinto, Maicon J. Hillesheim, Francisco C. de Araújo, Alexandre B. Santos, Delfim Soares

**Affiliations:** 1 Department of Electrical Engineering, UFJF, Juiz de Fora 36036-900, MG, Brazil; delfim.soares@ufjf.edu.br; 2 Department of Electronics, Federal Center for Technological Education of Rio de Janeiro, CEFET-RJ, Rio de Janeiro 20271-110, RJ, Brazil; milena.pinto@cefet-rj.br; 3 Faculty of Exact and Technological Sciences, UNEMAT, Sinop 78555-000, MT, Brazil; maicon@unemat-net.br; 4 Department of Civil Engineering, School of Mines, UFOP, Ouro Preto 35400-000, MG, Brazil; dearaujofc@ufop.edu.br; 5 Department of Structural Engineering, UFJF, Juiz de Fora 36036-900, MG, Brazil; alexandre.bessa@ufjf.edu.br

**Keywords:** photogrammetry, image displacement, point cloud, the Poisson’s method, displacement-surface fitting

## Abstract

This research employs displacement fields photogrammetrically captured on the surface of a solid or structure to estimate real-time stress distributions it undergoes during a given loading period. The displacement fields are determined based on a series of images taken from the solid surface while it experiences deformation. Image displacements are used to estimate the deformations in the plane of the beam surface, and Poisson’s Method is subsequently applied to reconstruct these surfaces, at a given time, by extracting triangular meshes from the corresponding points clouds. With the aid of the measured displacement fields, the Boundary Element Method (BEM) is considered to evaluate stress values throughout the solid. Herein, the unknown boundary forces must be additionally calculated. As the photogrammetrically reconstructed deformed surfaces may be defined by several million points, the boundary displacement values of boundary-element models having a convenient number of nodes are determined based on an optimized displacement surface that best fits the real measured data. The results showed the effectiveness and potential application of the proposed methodology in several tasks to determine real-time stress distributions in structures.

## 1. Introduction

Deformation monitoring in large structures, such as slopes and dams, requires constant inspection [1,2,3], as failures in these types of structures may cause severe environmental, social, and economic damages to the affected areas, including the straightforward risk to people’s life [4,5]. As a matter of fact, it is obvious that this class of engineering problem is most relevant, and to cite catastrophic accidents with tragic consequences for the communities in the affected areas, we mention the collapses occurring in the Fundão [5] and Brumadinho tailings dams in Brazil [6], respectively, in 2015 and 2019, which are definitely among the largest environmental disasters worldwide. It is worth noting that concerning large structures, nonconventional ways to measure strains and the associated stress states may also constitute insightful tools for the general safety evaluation of general structures, such as bridges undergoing seismic waves [7] or strong wind vortexes [8]. In this sense, possible changes in the surface of the structures can be regularly measured in order to estimate, in the end, their stability and safety. This is an especially suitable procedure when dealing with structures not easily accessible by fieldworkers. A challenge, though, in these techniques is to devise calculation strategies that may estimate stresses with reasonable accuracy. Particularly in the catastrophic collapse of the Brumadinho tailings dam, satellite monitoring has been conducted by Gama et al. [6] to detect ground deformation patterns along the downstream slope face of the dam prior to its failure. In fact, these techniques provide a qualitative assessment of the risk based on the observation of surface motions of the structures but not based on stress calculations.

The idea of using photogrammetry to measure mechanical quantities during deformations in structures was first introduced by Peters and Ranson in the 1980s [9]. Since then, photogrammetry and digital image correlation have matured and been applied to the field of experimental solid mechanics [10], aerospace industry [11], 3D shape measurement [12], civil engineering and bridge inspection [13] (for a comprehensive review, the work of Baqersad et al. [14] is suggested). Photogrammetry has also been applied in association with numerical modeling techniques (usually the (usually the finite element method) ) to analyze different types of structures; however, in these cases, they have been mostly applied to generate accurate geometric models for the subsequent evaluation of their condition states by means of numerical analysis [15,16,17]. Recently, remote measuring procedures have also been applied to determine displacements in structures [18,19,20,21], providing further advances in the field of non-destructive evaluations and structural health monitoring. In this work, all these aspects are combined and photogrammetry is applied, in conjunction with the boundary element method, so that the geometric model and its surface displacements may be determined, considering image processing techniques, and internal displacements and stresses may be evaluated in accordance with these pre-determined displacement boundary values, allowing to better monitor a structural system, in real-time.

Recently, many different methods have been applied to inspect structures, such as light detection and ranging (LIDAR), 3D Cameras, and Structure From Motion (SfM) [22,23,24]. Each method has advantages and limitations for structural assessment. The most commonly used techniques for detecting possible deformations in structures have taken into account control points (CP). In these techniques, specialists are responsible for defining the positioning of these CPs, and the changes can only be calculated in the regions where the sensors are placed [25]. In contrast, the use of techniques based on laser scanning and photogrammetry allows the monitoring of large areas [26].

A very well-known non-contact optical technique for 3D deformation measurements is the digital image correlation (DIC). This technique works by using a series of image pairs captured from two different views of the object in order to locate and track a given set of surface points during the deformation. Then, this method needs a random pattern of dark and light features to be provided into the object surface to find the best match between corresponding points in the two images. This correspondence is performed by comparing the local greyscale distribution of square pixel subsets based on the normalized cross-correlation coefficient [10,27]. This work also employs a non-contact optical technique. The proposed methodology uses the image displacement technique to generate the point clouds to define the 3D surface, then a Poisson method for surface reconstruction. By extracting the triangular meshes from the point clouds, the boundary element method (BEM) is applied to analyze the model.

Photogrammetry reconstructs three-dimensional surfaces using a sequence of images based on a computer vision technique called structure from motion [28,29,30,31,32,33]. This technique only requires a camera to be applied in the inspection task, which may drastically reduce the costs. Note that this is not the technique used in the experiment. Furthermore, the reconstructions generated by the SfM technique also provide information about their texture. In this sense, computer vision techniques are extensively used for monitoring the structures’ deformation. Note that SFM can yield comparable point clouds to LIDAR, and the algorithms of SfM are becoming computationally efficient [34,35]. Unlike traditional techniques, that is, displacement sensors, they are less costly and can monitor larger areas [36]. Thus, in the literature, different works have applied techniques based on images for this purpose. In [37], the author’s used a single camera configuration for a deformation monitoring system. A calibration pattern, placed close to the target’s location, was used to extract the camera’s intrinsic parameters. Through these parameters, the translation and rotation of the target were calculated. To avoid using physical markers placed on the surface to be monitored, the author’s of [38] used virtual markers. They used SIFT [39] features as virtual markers and, through them, tracked the occurred deformations.

A similar approach was developed in [40]. In their work, the author’s used commercial cameras to monitor structural deformation. However, unlike [38], this work used Harris corner features and the Kanade–Lucas–Tomasi (KLT) technique to track the movement of features. Then, the maximum likelihood estimation sample consensus (MLESAC) algorithm was used to remove outliers. The author’s of [41] used optical flow techniques to track deformations in an earthquake simulation configuration. In addition, to deal with adverse conditions, the author’s employed techniques for video stabilization and image denoising. Several other studies used visual techniques in the literature [42,43,44,45,46,47,48]. For more detailed information regarding image-based deformation monitoring, the author’s refer to [36]. Other methods, such as the use of LIDAR, have also been proposed in the literature. Works such as [49,50,51] used these techniques to inspect structures and monitor deformation. These methods showed good results. However, the use of a regular camera is cheaper and lighter than LIDAR, allowing its use along with modern inspection techniques, such as Unmanned Aerial Vehicles UAVs [52]. Moreover, traditional cameras can capture 3D displacements in thousands of points without any special mark, as presented in [29]. This approach is advanced compared to total stations or 3D LIDAR. While the first depends on fixed ground targets, the latter relies on complex and less reliable 3D descriptors to find the displacements.

In order to apply the above-mentioned visualization techniques to estimate failure in structures, an additional technique has to be used to evaluate the stress distribution occurring in them associated with the displacement fields photogrammetrically captured on their surface. In the structural engineering community, two major numerical methods have been employed for the analysis of structural systems: the finite element method (FEM) [53,54] and the boundary element method (BEM) [55,56,57,58]. The FEM is by far the method currently most applied in developing structural projects, and nowadays, a series of powerful FE-based commercial packages are available to structural engineers, e.g., ANSYS [59], SAP2000 [60], and ABAQUS [61]. However, being a domain discretization method, the FEM presents drawbacks in modeling open-domain problems, as regions extending to infinite must be truncated. This is typically the case of soil–structure interaction problems. On the other hand, BEM formulations are based on the boundary integral representation of the solution for a given problem. Thus, the entire description of its solution in its whole definition domain depends exclusively on knowing all its boundary values. No discretization of the domain is then required to approximate the field variables. Furthermore, the boundary integral representation of the problem response describes exactly the solution’s behavior at the boundary parts lying at infinite, which do not need to be discretized as well [55]. In general, once the response has been determined at the whole boundary of a deformable solid under loading, the needed solution at points in its interior may be then promptly calculated by invoking again the available boundary integral representation of the sought-after response [55]. In References [62,63,64,65], advances concerning integration and solution algorithms specialized for 3D boundary-element formulations are discussed. In conclusion, for 3D elastostatic applications, the following main advantages of the BEM over the FEM can be highlighted: (i) the BEM provides more accurate results than the FEM considering equivalent element-size discretizations since it considers a more elaborated mathematical construction; (ii) the BEM usually provides more efficient analyses than the FEM, since reduced systems of equations are then regularly obtained, and only the boundary of the model needs to be discretized; (iii) the BEM allows simpler analyses to be carried out since it enables simplified discretization procedures and, consequently, requires fewer data to be managed. Notice that the use of the BEM along with the image displacement technique to estimate stresses inside a solid, which is being proposed in the present paper, is a quite convenient strategy, as the displacement fields captured by the image displacement technique on the structure surface are precisely part of the solution that needs to be entered into the BE code for stress calculation.

In this work, we demonstrate the use of long-period fiber gratings (LPFGs) as a fiber Bragg grating alternative in quasi-distributed strain sensing. To validate the use of multiple LPFGs in multi-point structural strain sensing, we placed four sensors in an aluminum beam, measured the corresponding strains, and compared them with the proposed techniques.

### 1.1. Main Contributions

This research work proposes an innovative image-based technique to estimate a real-time displacement distribution occurring on 3D surfaces when they move from one deformation configuration to another. In this process, an image displacement technique is applied to generate point clouds as well as to reconstruct the 3D surfaces, which are based on triangular 3-node boundary element meshes extracted from the corresponding point clouds. The captured displacement fields are then promptly entered into a boundary-element code to estimate the stress distributions experienced by the structure or solid during its deformation. To observe the performance of the strategy, tests were carried out on an aluminum bar subjected to bending and having displacements photogrammetrically measured in a part of its boundary. In general, this research presents the following main contributions:

- To demonstrate the advantages of using photogrammetrical approaches to determine orthogonal displacements in objects;
- Application of the boundary element method (BEM) to evaluate stress distributions based on optimized displacement surfaces;
- To demonstrate the application of image displacement along with BEM techniques to estimate real-time stresses in solids and structures;
- Validation of the results by comparing strain measures in an aluminum bar obtained by using long-period grating (LPG) optical fiber sensors and the proposed strategy.

### 1.2. Organization

The remainder of this work is organized as follows. Section 2 details the proposed deformation tracking methodology and its mathematical foundations. Section 3 presents the experiments with a proper discussion of the results. The concluding remarks and future work are conducted in Section 4.

## 2. Image-Based Approach for Deformation Estimation

**Figure 1:**
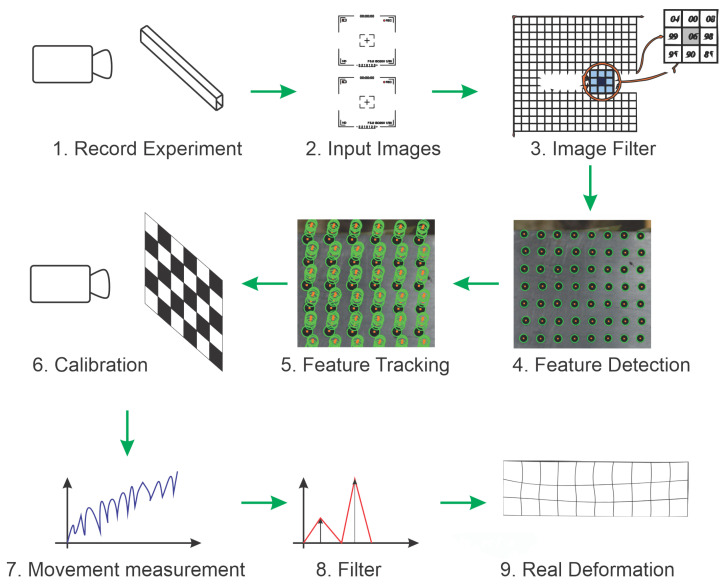
Representation of the proposed methodology for calculating displacement in structures.

The first part of the proposed methodology is summarized in Figure 1. A computer vision technique is applied to estimate displacement in different parts of an analyzed object. The first images from the object to be analyzed are captured over time using a single camera. In step 2, the images are stored and then filtered in step 3. The filtered images undergo feature detection in step 4. Those features will be used to track deformation along time, as represented in step 5. In this stage, the position of each feature in each image is stored in an organized fashion. Note that this information already represents the movement of different parts of the structure in the camera space. Therefore, the camera calibration process (step 6) is used to estimate homograph matrices and transform the feature positions to real-world measurements. In step 7, sets of points data are combined to allow movement estimation for each object’s region under deformation. The movement information is filtered in step 8 to eliminate errors. At the end (step 9), data are combined into a displacement vector for each part of the analyzed object.

The above-described process is applied here, considering an aluminum bar with known dimensions, which is considered the object of interest for monitoring displacements. The proposed method for deformation calculation uses a fixed camera and a pattern painted on the surface to be monitored. The chosen pattern consists of an M×N grid of circles with radius *r*, and the distance between each circle is δ. This pattern was used because it is easy to be constructed, and there are well-established methods for detecting it in an image. In this sense, circles with known sizes and positions were painted on this bar.

An external force, F, will deform the bar over time, and the deformations that occur will be calculated from the detection of the painted circles in the captured images. Thus, t=0 is considered the instant before the force’s application, and t>0 when the force is already active on the bar.

Considering that a global coordinate system originates from the circle located in the upper left corner, the position of the circle located in the *i*-th row and *j*-th column is given by $P\left(i,j\right)={\left(\delta j,\delta i,0\right)}^{T}$. Where δ is the distance from adjacent circles in millimeters. Since the bar is flat, the *Z* coordinate position was considered zero for all circles.

The deformation is calculated at the positions where each circle of the pattern was painted. Thus, initially, it is necessary to detect each circle in the image. To this end, the Hough transform can be used. This transform is widely used to detect curves in parametric form.

It is possible to detect the circles’ position of the images captured from the bar using the circular Hough transform. This is shown in step 4 of Figure 1. As the position of the circles in global coordinates is known, it is possible to map the coordinates of the circles detected by the Hough transform to the proposed global coordinate system through a homography relationship, which is presented in Equation (1). Notice that the homograph matrices are estimated in the camera calibration process (step 6).

(1) $$\lambda \left[\begin{array}{c} x_{i,j}\\ y_{i,j}\\ 1 \end{array}\right]=H\left[\begin{array}{c} \delta j\\ \delta i\\ 1 \end{array}\right]$$

**Figure 2:**
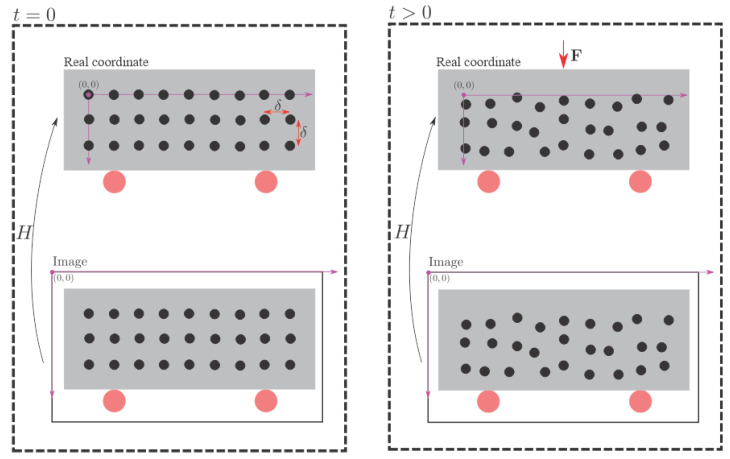
Proposed methodology for calculating displacement in structures.

In Equation (1), $x_{i,j}$ e $y_{i,j}$ are the coordinates *x* and *y* of the circle located in the *i*-th row and *j*-th column. The variable λ is a scale factor. Note that it takes at least four points to find the *H* matrix. The matrix *H* is calculated in t=0, that is, before the bar undergoes any deformation caused by the external force. For t>0, it is still possible to detect the circles in the image using the Hough transform. However, it is not possible to know the circles’ arrangement in the global coordinates. In this way, it is not possible to compute the *H* matrix during the experiment. However, the matrix calculated in t=0 is still valid in case the camera remains fixed throughout the experiment. Thus, the new circles’ position in the global coordinates can be found only by multiplying the matrix *H* by $p\left(i,j,t\right)={\left(x_{i,j,t},x_{i,j,t},1\right)}^{T}$. Therefore, the circle’s displacement located in the *i*-th row and *j*-th column at the time t>0 is given by the Equation (2), where, in the image, $x_{i,j}$ e $y_{i,j}$ are the coordinates *x* and *y* of the circle located in the *i*-th row and *j*-th column at time *t*. Figure 2 illustrates the proposed methodology for calculating displacements in two dimensions. Note the use of matrix *H* in the figure.

(2) $$d\left(i,j,t\right)=H\left[\begin{array}{c} x_{i,j,t}/\lambda \\ y_{i,j,t}/\lambda \\ 1/\lambda \end{array}\right]-P\left(i,j\right)$$

When the monitored structure is deformed, the positions of pattern circles are modified as well. However, as the relative position between the camera and the pattern plane remains fixed, the homographic matrix does not change. Therefore, after detecting the new location of the pattern circles in the image using the circular Hough transform, it is possible to calculate the circles’ position in the world coordinate system. Then, the new circles’ position in the world coordinate system is compared with their initial position, thus obtaining the deformation. In this way, displacements can be calculated for each new frame captured during the experiment.

To track the structural deformation, two approaches were considered. The first one was to capture an image before and after the deformation process. The second one was to record the entire deformation process. It was observed that constant monitoring via video enabled the deformation calculation frame by frame. Thus, we were able to mitigate possible random variations between close frames. Since the second approach allows for the filtering of this random noise, it was the chosen approach in this work. Two filters were applied to the obtained deformations, denoted by temporal and spatial filters. They are described in the following.

Note that the method was applied by using markings in the bar. However, this may not be possible for every real-world structure. In this sense, other techniques such as image matching using feature descriptors such as SIFT, SURF, ORB, or [29] can be used. Image displacement methods, such as this one, can also be used along with SfM [66] inspections where a single moving camera is used to build a colored 3D model of the environment.

### 2.1. Spatial Filtering

Spatial Filtering consists of smoothing the displacement captured for the circle in (i,j) using the displacements of neighboring circles. This can be done through a kernel applied to the displacement matrix d(i,j,t). The application of the smoothing kernel *G* of size $n_{v}$ × $n_{v}$ can be verified in Equation (3), which represents the process of sweeping the Kernel function G over the measurement space.

(3) $$d_{esp}\left(i,j,t\right)=\frac{\sum_{u=-n_{v}}^{n_{v}}\sum_{k=-n_{v}}^{n_{v}}G\left(u,k\right)d\left(i+u,j+k,t\right)}{\sum_{u=-n_{v}}^{n_{v}}\sum_{k=-n_{v}}^{n_{v}}G\left(u,k\right)}$$

### 2.2. Temporal Filter

Note that small changes in lighting in the environment can modify the Hough transform’s circle detection capacity during the capture of images in the experiment. Thus, small-displacement variations can be verified over time. One way to mitigate this effect is to apply a moving average filter to the found displacements. Equation (4) shows the application of the moving average filter for the *i*-th row and *j*-th column of the circle, where *M* is the width of the moving average filter.

(4) $$d_{temp}\left(i,j,t\right)=\frac{1}{M}\sum_{k=0}^{M-1}d\left(i,j,t+k\right)$$

The use of combined spatial and temporal filters smooths the measurements and attempts to eliminate camera and image classification errors such as outliers. It is important to highlight, however, that other techniques could also be applied to remove anomalies, such as, for instance, the maximum likelihood estimation (MLE) [67,68].

### 2.3. Boundary Element Method

In the BEM, displacements and stresses at inner points of a solid are expressed in terms of the following boundary integral equations:

(5) $$c_{ik}(\xi )u_{i}\left(\xi \right)+\int_{\Gamma }p_{ik}^{*}\left(x,\xi \right)u_{i}\left(x\right)d\Gamma \left(x\right)=\int_{\Gamma }u_{ik}^{*}\left(x,\xi \right)p_{i}\left(x\right)d\Gamma \left(x\right)+\int_{\Omega }u_{ik}^{*}\left(x,\xi \right)b_{i}\left(x\right)d\Omega \left(x\right)\;,\;$$

(6) $$\sigma_{ij}(\xi )+\int_{\Gamma }u_{lij}^{*}\left(x,\xi \right)p_{l}\left(x\right)d\Gamma \left(x\right)=\int_{\Gamma }p_{lij}^{*}\left(x,\xi \right)u_{l}\left(x\right)d\Gamma \left(x\right)+\int_{\Omega }u_{lij}^{*}\left(x,\xi \right)b_{l}\left(x\right)d\Omega \left(x\right)\;,\xi \in \Omega \left(x\right)\;,$$

where $\xi =(\xi_{x},\xi_{y},\xi_{z})$ is the inner point (source point) where displacements and stresses should be calculated in the 3D region, x=(x,y,z)∈Ω(x) is the field point, $u_{i}$, $p_{i}$, and $\sigma_{ij}$ denote, respectively, displacements, boundary tractions (forces per unit area), and stresses, $b_{i}$ represents body (volume) forces, and $c_{ik}$ is the jump term. The terms $u_{ik}^{*}$, $p_{ik}^{*}$, $u_{lij}^{*}$, and $p_{lij}^{*}$ are the known elastostatic fundamental kernels involved in the integral representation of general elastostatic solutions in deformable solids (see Love [69]), which embed the constitutive material law and are explicitly given by

(7) $$\begin{array}{c} \begin{array}{ccc} u_{ik}^{*}\left(x,\xi \right) & \;\;\;\;=\;\;\;\; & \frac{1}{16\pi (1-\nu )Gr}\left[\left(3-4\nu \right)\delta_{ik}+r_{,i}r_{,k}\right]\\ p_{ik}^{*}\left(x,\xi \right) & \;\;\;\;=\;\;\;\; & \frac{-1}{8\pi \left(1-\nu \right)r^{2}}\left\{\left[\left(1-2\nu \right)\delta_{ik}+3r_{,i}r_{,k}\right]\frac{\partial r}{\partial n}-\left(1-2\nu \right)\left(r_{,i}n_{k}-r_{,k}n_{i}\right)\right\}\\ u_{lij}^{*}\left(x,\xi \right) & \;\;\;\;=\;\;\;\; & \frac{1}{8\pi \left(1-2\nu \right)r^{2}}\left[\left(1-2\nu \right)\left(r_{,i}\delta_{lj}+r_{,l}\delta_{ij}-r_{,j}\delta_{li}\right)+3r_{,l}r_{,i}r_{,j}\right]\\ p_{lij}^{*}\left(x,\xi \right) & \;\;\;\;=\;\;\;\; & \frac{G}{4\pi \left(1-2\nu \right)r^{3}}\left\{3\frac{\partial r}{\partial n}\left[\left(1-2\nu \right)r_{,j}\delta_{li}+\nu \left(r_{,l}\delta_{ij}+r_{,i}\delta_{lj}\right)-5r_{,l}r_{,i}r_{,j}\right]\right.\\ & + & \left.3\nu \left(r_{,j}r_{,l}n_{i}+r_{,j}r_{,i}n_{l}\right)+\left(1-2\nu \right)\left(3r_{,l}r_{,i}r_{,j}+n_{l}\delta_{ij}+n_{i}\delta_{lj}\right)-\left(1-4\nu \right)n_{j}\delta_{li}\right\} \end{array}\;. \end{array}$$

Notice that Γ(x) denotes the whole boundary of the solid under analysis. The boundary integral Equation (5) tells us that once the boundary displacement and traction fields are known in the whole boundary Γ of a solid, the determination of all desirable variables in its interior is straightforward. This is the basic idea of the BEM, which takes the boundary integral Equation (5) as a starting point for deriving the numerical method. Thus, discretizing the boundary of the solid with a convenient number of boundary elements, ne, over which one assumes that the variables $u_{i}$ and $p_{i}$ vary according to a given polynomial, e.g., linear or quadratic, an algebraic system of equations of the form

(8) Hu=Gp+b

is obtained, where H and G are the BE system matrices, and u, p and b are, respectively, the vectors storing the displacements, tractions, and the body force contribution at the nodes of the boundary element mesh. In a regular boundary value problem (BVP), part of the displacements and tractions are prescribed, and part of them are unknown. Thus, by reordering the algebraic system of equations so as to move all the unknown values to the left-hand side of the system and to leave the prescribed values on its right-hand side, the unknown variables may be determined by solving the resulting system of equations.

In References [55,70,71], basic details on boundary integral formulations for elasticity problems are addressed, including a discussion on the derivation of the involved fundamental kernels. In fact, with the more recent advances brought about by scientists and engineers in applying the boundary element method to general physical problems, this method has been definitely established as a relevant analysis tool in engineering [72,73]. For a deeper insight into advanced techniques embedded in the construction of general boundary-element codes, which necessarily must take into account special integration algorithms for dealing with the singular kernels, References [62,63,64,65] may be considered. Solution techniques for the resulting system of algebraic equations, discussed in these papers, are also fundamental for practical applications of the BEM, mainly in cases where heterogeneous materials are present and the resulting systems of equations are high order.

It is worth mentioning that, unlike conventional boundary-value problems, in the particular strategy proposed in this paper for the real-time evaluation of stresses, a different type of boundary condition is available, namely the one in which both displacements and tractions are known. This is exactly the case of the free surfaces of the body where tractions are prescribed, and the displacements are obtained by employing the image displacement technique. In this case, the system of equations is reduced by condensing out the captured displacements.

**Figure 3:**
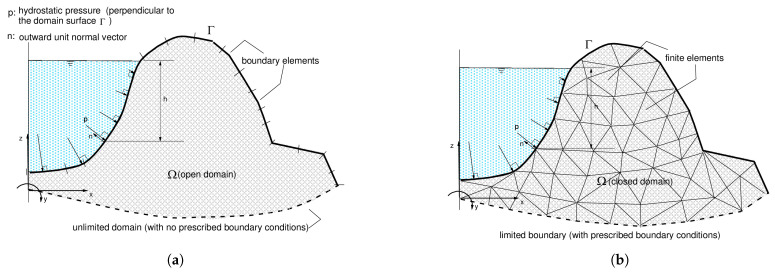
Water dam (**a**) BE model and (**b**) FE model.

Specifically to highlight the relevance of applying the boundary element method, instead of the finite element method, along with the SFM-based imaging technique as well as with any other imaging technique, one considers the generic models depicted in Figure 3. Assuming generically that the whole boundary Γ of the boundary-element model (see Figure 3a) is subdivided into a part $\Gamma_{1}$ with prescribed displacements, ${\bar{u}}_{1}$, and unknown boundary tractions, $p_{1}$, and another part $\Gamma_{2}$ with both known displacement, ${\bar{u}}_{2}$, and tractions, ${\bar{p}}_{2}$, the system of Equations (8) may be written in partitioned form as

(9) $$\left[\begin{array}{cc} H_{11} & H_{12}\\ H_{21} & H_{22} \end{array}\right]\left\{\begin{array}{c} {\bar{u}}_{1}\\ {\bar{u}}_{2} \end{array}\right\}=\left[\begin{array}{cc} G_{11} & G_{12}\\ G_{21} & G_{22} \end{array}\right]\left\{\begin{array}{c} p_{1}\\ {\bar{p}}_{2} \end{array}\right\}+\left\{\begin{array}{c} b_{1}\\ b_{2} \end{array}\right\}\;,$$

so that the boundary-element system of Equation (9) is reduced to

(10) $$G_{11}p_{1}=H_{11}{\bar{u}}_{1}+H_{12}{\bar{u}}_{2}-G_{12}{\bar{p}}_{2}-b_{1}\;,$$

where the only unknown variables of the problem are the traction values stored in $p_{1}$. Thus, the final BE model has considerably fewer degrees of freedom in comparison to the initial one. In fact, the larger the $\Gamma_{2}$ boundary is (because the imaging technique can be applied to estimate the displacement fields of a large surface’s part with known boundary tractions), the more efficient the proposed process will be. Contrarily to that, as one can easily infer from the generic finite elements (FE) model depicted in Figure 3b, although some reduction on the total number of degrees of freedom is attained by imposing known displacements and tractions on the boundary Γ of the FE model, the unavoidable discretization of the whole domain will still imply much higher order of the final FE system of equations. This is especially the case of problems defined in open domains, such as in geotechnical problems involving the soil, where FE discretizations have to take into account large portions of the (infinite) domain to conveniently simulate the behavior of the far-field response of the real problem (see Figure 3b). In the BE model (see Figure 3a), the boundary lying at infinity is simply disregarded, as the regularity conditions are perfectly satisfied by the corresponding boundary integral formulations (see [55]).

## 3. Results and Discussion

### 3.1. Experiment Setup

**Figure 4:**
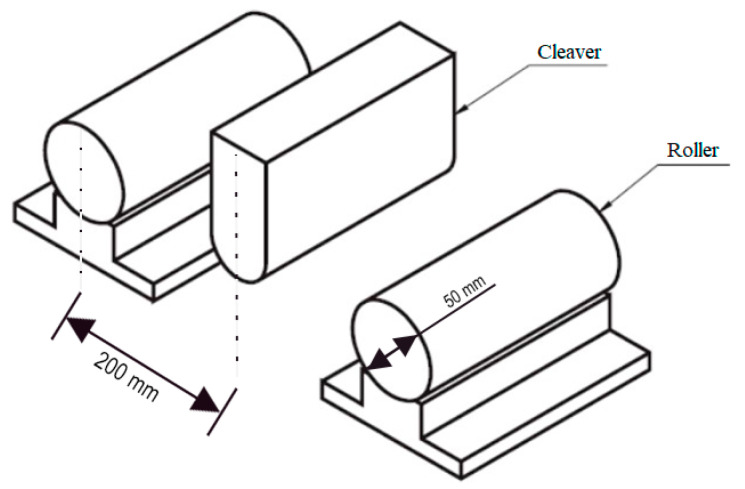
Experiment schematic.

**Figure 5:**
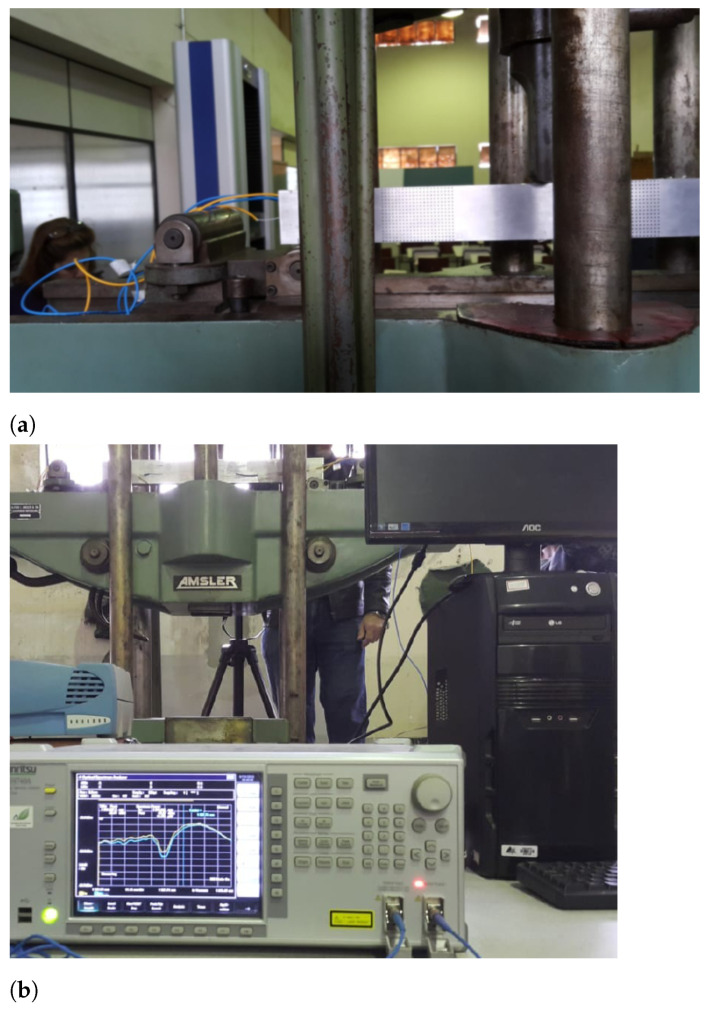
Experiment configuration (**a**) Camera positioned in front of the experiment (**b**) Aluminum bar under 610 kg effort.

To verify the strategy, a 3-point bending test (a schematic representation of the supports of the model is depicted in Figure 4) is performed on the aluminum bar shown in Figure 5, which is 539.5 mm long, 18.7 mm wide, and 63.13 mm high, and was subjected to upward lateral displacements on both supports at the bar ends. The LPG optical fiber sensors were positioned in different places in order to measure the strains resulting from the bending of the bar. It is important to highlight that after being installed on the bar, the optical fiber sensors were calibrated. The bar, made of aluminum with elasticity modulus E=69 GPa and Poisson’s ratio ν=0.321, was placed between two supports, as shown in Figure 4. In addition, a grid of black circles was painted where the distance from adjacent circles is 10 mm.

For this experiment’s configuration, the horizontal displacements expected for the bar are very small, and the resolution of the camera is limited. Thus, only vertical displacements could be captured using this methodology. The images’ capture was performed by video with the acquisition of 30 frames per second with a resolution of 1920 × 1080 px. Note that a camera Nikon 3200 was positioned in front of the bar to monitor the circle’s displacements. A representation of the camera view is shown in Figure 5a; thus, it is only a representation of the camera view at one point during the experiment where the camera is quite close. The entire test setup is presented in Figure 5b.

**Figure 6:**
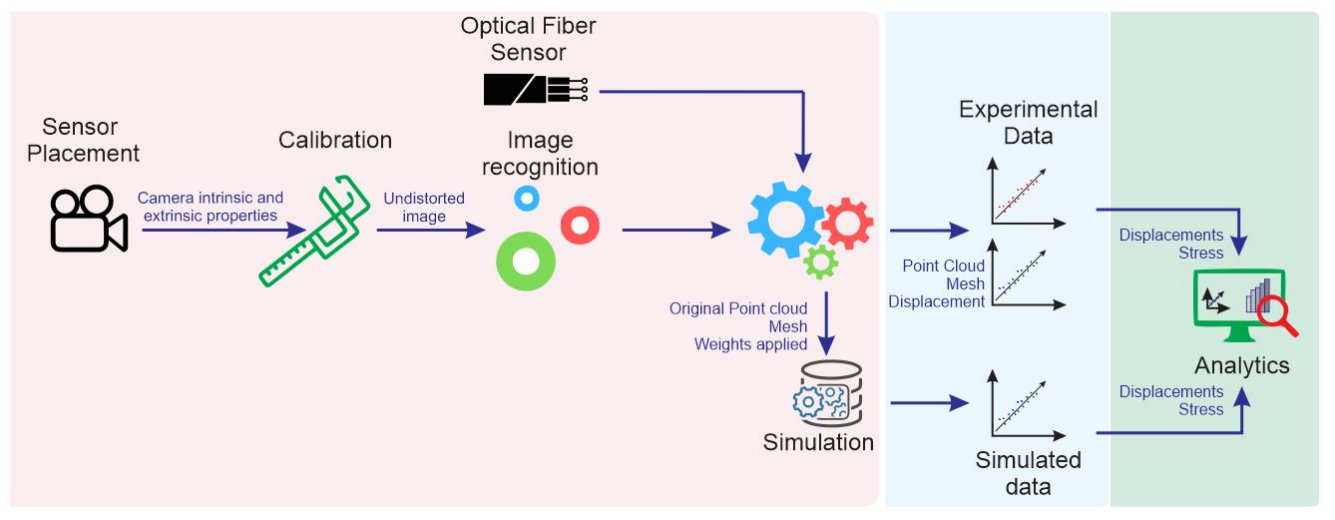
Experimental workflow.

The experimental process is composed of three main steps, as represented in Figure 6. In the first step, the experimental setup, which is explained in this section, is regarded, and sensors are placed and calibrated. Then, the experimental data are gathered using the many sensors placed at the specimen. Simultaneously, in this second step, a simulation is performed using the parameters obtained during the experimental setup. Finally, in the third step, the data gathered from all these sources are analyzed.

### 3.2. Optical Fiber Sensors

As it typically happens in a 3-point bending test (see Figure 5), the force *F* acting at the middle cross-section of the beam (bar) causes it to bend. Thus, in case this force *F* acts downward, the superior fibers in the beam will experience compression while the inferior ones will experience tension. The regions of the bar under compression (the superior one) and tension (the inferior one) are separated by an internal surface in which zero normal strains and stresses (transition plane) are observed. This is the neutral surface of the beam, and its intersection with a given cross-section defines its neutral axis, which generically lies on the barycentric point of the cross-section and corresponds to the axis around which the cross-section rotates. This explanation above gives a short insight into the general physical aspects of the bending phenomenon considered in our experiment. A deeper discussion on it, which provides a complete mathematical definition of the involved physical variables, such as strains and stresses, is found in [74].

In our experiment, in addition to using the photogrammetric technique we are proposing in this study, we also conduct strain measurements by using LPG fiber optical sensors. For that, the sensors S021, S022, S023, and S024 are glued at the bar surface, and the load level imposed on the beam is limited to values for which the maximum normal stresses do not exceed the aluminum yield stress, i.e., the bar is behaving physically elastic. After being installed on the bar, the sensors are calibrated. For this purpose, metal washers with known mass are used, and these washers are measured using a precision scale. It is possible to calculate the strain that the bar undergoes at each sensor’s position using the masses’ values. Table 1 presents the relation between the sensors strain Δλ (nm) and the strain caused on the bar ϵ.

To calibrate the bar measurements, the movements of the circles close to the sensor position are combined, and this information is used to produce an associated correction matrix. This matrix represents a correction from pixel information to real-world data.

These results can be seen as a baseline for deformation analysis. They can be used to verify simulated and measured displacements built from photogrammetry.

### 3.3. Photogrammetry Experiment

**Figure 7:**
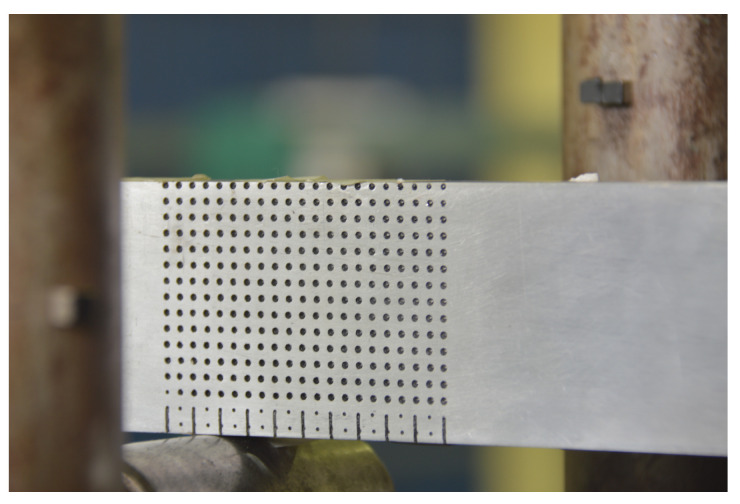
Circles monitored by the proposed methodology.

The computer vision part of the methodology was implemented in MATLAB. The experiments consist of image matching processes, feature extraction, matching visual descriptors, and matching three-dimensional points. A camera was placed in front of the experiment bar to monitor the displacements of the black circles, as shown in Figure 7. In addition, for reference, the circles were numbered from 1 to 270.

The deformations expected for an aluminum bar with the same specifications as the bar used and under the same conditions of the experiment were simulated to obtain ground truth. The results obtained by the proposed methodology were compared with the simulations. Note that small variations in lighting can impair the calculation of the circles’ positions over time. There, spatial and temporal filters were implemented together to mitigate this problem.

**Figure 8:**
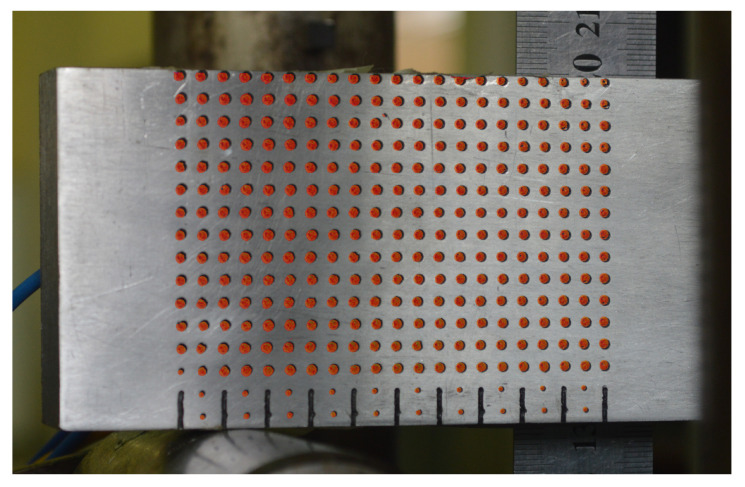
Overlay points positions.

The next step is to use the movement of the other elements in the picture to determine parts that are not related to the bar deformation. After this correction, the true movement of the bar is obtained. Figure 8 shows this result, where the red circles overlayed on top of the picture shows the real movement generated by the bar deformation. It is important to mention that only a small section of the bar is monitored for deformation. This area at the extremity of the bar allows a good method of visualization. The circles were placed in defined known distances to allow measurement validation. Note in this figure that real movement due to deformation is only a fraction of the total movement of the bar. Notice that as a single fixed camera is used, it is impossible to reconstruct a 3D point cloud. However, before the experiment, the performed camera calibration allows estimating a 2D point cloud related to the experiment’s real dimensions.

**Figure 9:**
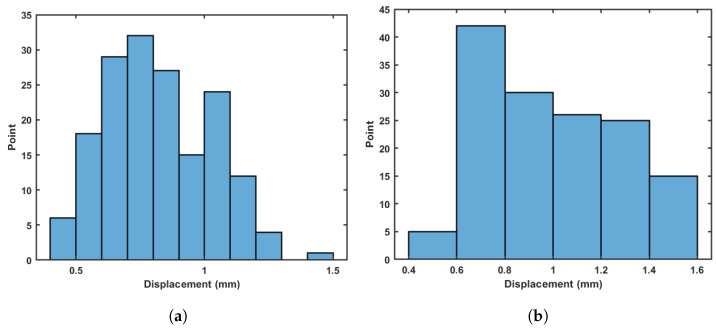
Histogram of displacements (**a**) Simulated (**b**) Measured.

Using these new displacements, it is also possible to build a histogram of movement only due to bar deformation. Figure 9a shows this result. Observe that this movement is very small in comparison with the histogram of Figure 9b. Note that the error due to photogrammetry will be double the value calculated previously (i.e., ±0.2 mm) once the two measurements are combined to form this result.

**Figure 10:**
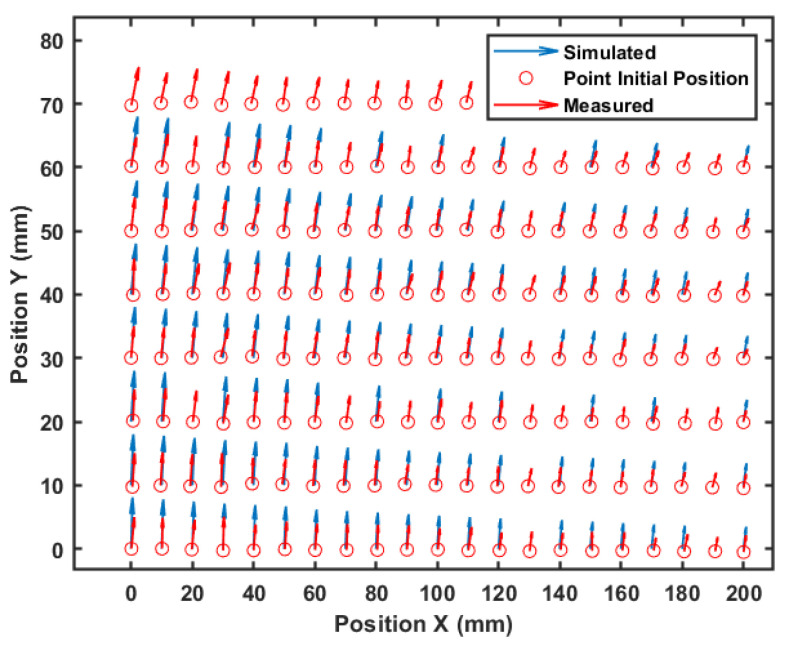
Movement direction for each point.

The histograms allow a quantitative visualization of the observed deformation. However, to visualize the movement direction of each point, Figure 10 shows the total displacement of each point. The arrow represents the movement direction, and its length gives an idea of its module. Note that in blue, the simulated values are close to the measured ones in red.

Next, to evaluate the proposed technique against point cloud-based methods, the author’s have reconstructed the bar surface and its point cloud representation from the experimental data by assuming that the camera is in a fixed, known position and using a second image along with SfM to produce a point cloud [75].

**Figure 11:**
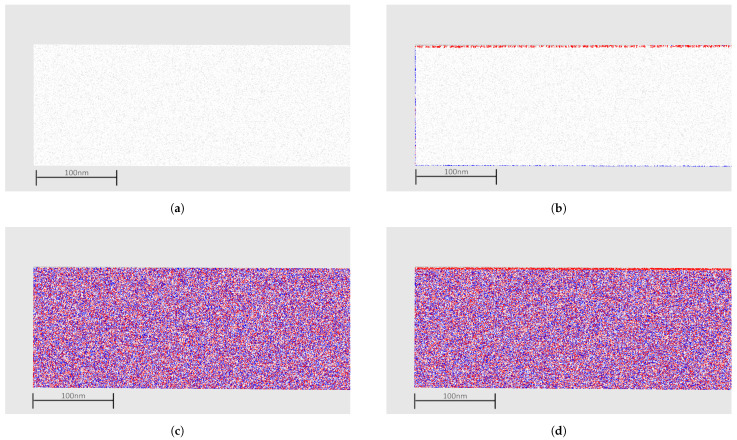
Distance estimation for a simulated point cloud (**a**) Surface points (**b**) C2C distance in Z axis (**c**) C2C distance in X axis (**d**) C2C distance in Y axis.

A simple method to determine displacements in point clouds is to take one point in the reference cloud, find the closest point in the second cloud, and estimate its distance. This method is often called Cloud to Cloud or C2C, and some implementations may estimate the local surface and calculate the distance to this surface. Figure 11a shows the simulated surface points to simulate a depth camera or LIDAR usage. A second surface after the application of forces is also reconstructed, and the distance between them is estimated.

In the figure, negative displacements are shown in red, and positive displacements (in blue) indicate no displacement. Note in Figure 11c,d that the distances are noisy between blue and red, indicating that there are no meaningful displacements in these axes. In addition, note in Figure 11b that displacement is visible but only at the borders of the image. This is related to how the distances are estimated and do not reflect reality; as seen previously, all points have a similar displacement. This is a strong advantage of the method proposed once all displacements are properly estimated.

### 3.4. Boundary-Element Analysis

**Figure 12:**
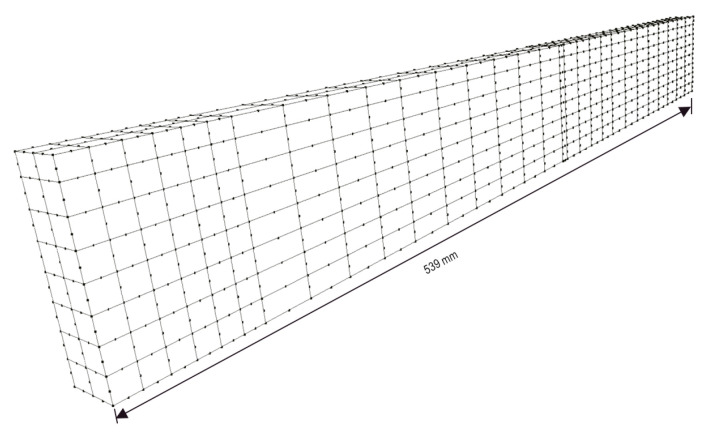
Perspective view of the BE model.

Particularly in the case of the present bending test, the boundary-element (BE) model depicted in Figure 12 has been employed for solving the beam. The mesh shown in Figure 12 contains 618 8-node quadratic boundary elements, having a total of 1838 nodes and 5514 degrees of freedom (DOF). To discuss the performance of the strategy proposed in this paper, two analyses were carried out.

**Figure 13:**
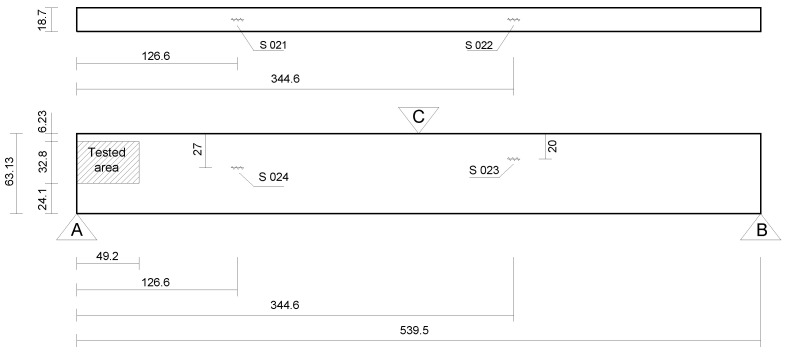
Schematic description of the bar model (length measures in mm).

**Figure 14:**
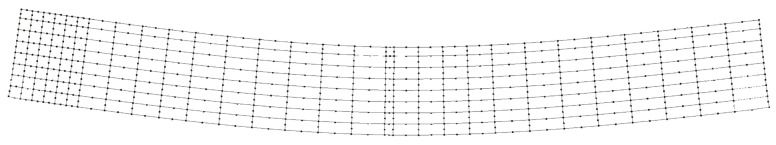
Lateral view of the deformed bar.

**Figure 15:**
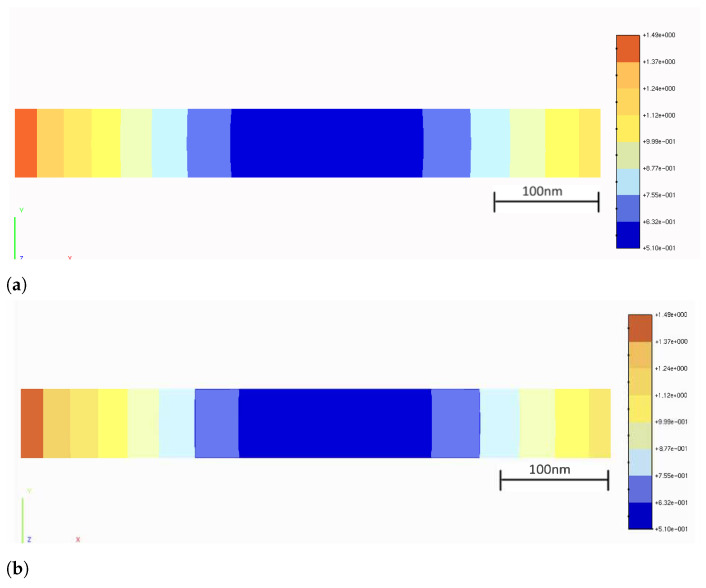
Vertical displacement component, $u_{y}$ (in mm), in the internal plane (**a**) BE analysis I: image displacement-measured displacement exclusively applied at the supports (**b**) BE analysis II: image displacement-measured data applied at the hatched area and supports.

**Figure 16:**
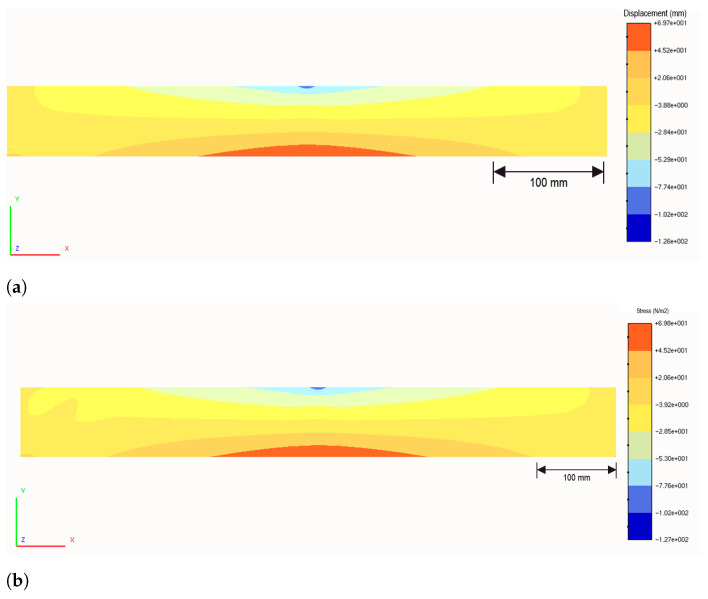
Normal stresses, $\sigma_{xx}$ (in MPa), in the internal plane (**a**) BE analysis I: image displacementmeasured displacement exclusively applied at the supports (**b**) BE analysis II: image displacementmeasured data applied at the hatched area and supports.

**Figure 17:**
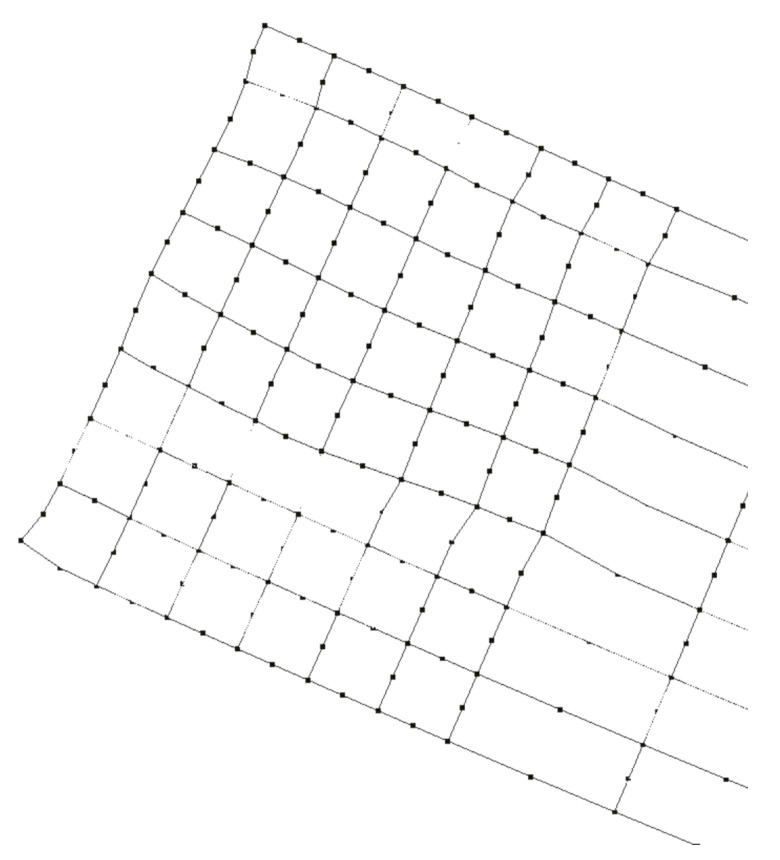
Deformation capture by image displacement.

In the first analysis (BE analysis I), only the displacements photogrammetrically measured at the three supports A, B, C of the bar (see Figure 13 and Table 2) were considered as prescribed boundary conditions, and the bar was solved by applying a homemade BEM computer code, capable of analyzing stresses in 3D solids. The aspect of the deformed bar at the final load level in the test (610kgf) is shown in Figure 14. The corresponding results in terms of displacements and stresses at an internal plane positioned at half the width of the beam are shown in Figure 15a and Figure 16a, respectively. In the second analysis (BE analysis II), in addition to the support displacements given in Table 2, the displacements measured on the hatched area by using the SFM technique and shown in Figure 10 were considered as well in the BE analysis. In fact, as the BE mesh corresponding to the hatched area has fewer nodes than the number of points considered in the experiment in that area (depicted in Figure 10), a 2D regression technique has been applied to project the photogrammetrically captured displacements on the adopted BE mesh. This process optimizes the displacement values at the nodes of the BE mesh based on the photogrammetrically measured values. In Figure 17, part of the deformed beam that includes the SFM measured displacement data is shown. In addition, the vertical displacement components and normal stress $\sigma_{xx}$ obtained in this analysis for the same internal plane mentioned above, at half the beam width, are presented in the color plots in Figure 15b and Figure 16b, respectively.

Finally, to validate the first results obtained with the proposed technique, the strain component $\epsilon_{xx}$ measured by employing optical fiber sensors at the positions S021, S022, S023 and S024 (see Figure 13) are compared to the corresponding ones determined by using the image displacement technique along with the BEM (BE analyses I and II described above). These results are presented in Table 3. As one sees, the strain values obtained by employing the optical fiber sensors and the combined image displacement/BEM technique agree with good accuracy, although the amounts of strain are very low order. Notice that higher normal stress values are observed on the superior beam surface, around the point *C* shown in Figure 13, in comparison to the inferior one. In fact, this happens because point *C* is where the load is applied, and the concentrated nature of the load brings about a considerable stress level increase in the region around it.

### 3.5. Analysis of Results

By evaluating the results presented in the last three sections, it is possible to observe few different aspects. First, it is possible to use photogrammetry methods to track objects’ features over time and thus monitor their movement. This is shown by comparing the photogrammetry measurements with simulated values for the experiment showing similar results. Those are also in line with the physical measurements made at the site.

It was also possible to validate the usage of photogrammetry methods in combination with BEM to estimate structure stresses. These results were also validated by using conventional LPG sensors to measure strains and associated stresses at selected points of the bar considered in the experiment. Nonetheless, it is worth mentioning that this experimental method may be applied online to obtain estimations of stresses in any solids and structures as long as the displacements may be satisfactorily measured at their surface. In fact, the apparent limitation of this method, arising from taking into account only measurements perpendicular to the camera view direction, may be easily overcome by using other photogrammetric techniques, such as stereoscopic vision or colored LIDAR or SfM, to produce 3D point information.

Furthermore, the camera calibration and resolution need to be carefully selected to allow precise measurements; any deformation in the image will likely produce wrong measurements. Another limitation is the physical markers usage. In the experiments, the author’s used these markers. However, for real-world experimentation, the usage of feature descriptors can allow the method used if there is enough texture in the images. In any case, even when deploying Lidar sensors, it would be hard to produce precise measurements and determine their proper directions if there is not enough texture in the data.

## 4. Conclusions and Future Work

This research work proposed an innovative and robust method to estimate displacement in monitored objects. The strategy combines a computer vision technique to estimate real-time displacement fields on a solid surface with the boundary element method (BEM), which can conveniently use photogrammetrically captured displacement fields to determine real-time stress distributions when a solid or structure experiences deformation. Herein, the image displacement technique is applied to generate the point clouds and to reconstruct the displacement-based 3D surfaces. In this paper, we presented the first results obtained by employing the proposed technique to measure real-time strains and stresses occurring during a 3-point bending test on an aluminum bar. As seen, by comparison with a strain measurement technique based on the use of optical fiber sensors, the image displacement-BEM-combined strategy was capable of accurately determining the normal strain components on given points on the surface of the aluminum bar. In fact, although the obtained amounts of strain are of very low order, both techniques (i.e., the one employing optical fiber sensors and the one considering combined image displacement/BEM procedures) provide very similar results, which are within a 2% margin of error. These results hint that the strategy is a promising technique that can be suitably employed for monitoring stress distributions on real structures.

As future works, one aims to develop mechanisms of capturing the displacement fields in larger structures so as to be able to construct efficient computational tools able to furnish real-time stress distributions and the corresponding safety level the structures undergo during their life. Computationally, the application of the BEM by condensing out the variables on the boundary parts with prescribed traction and photogrammetrically measured displacement fields is also relevant, as the global system of equations may be significantly reduced. This will be even more relevant for problems defined in infinitely extending regions, such as in soil–structure interaction problems. The author’s also intend to deploy the method for online use in a real structural inspection. In this work, fixed points were used for monitoring the deformation. In this sense, as future works, these points will also be replaced by feature descriptors estimated from an image.

## Figures and Tables

**Table 1:** Sensors and bar deformations.

S021		S022		S023		S024	
Δλ **(nm)**	ϵ	Δλ **(nm)**	ϵ	Δλ **(nm)**	ϵ	Δλ **(nm)**	ϵ
0	0	0	0	0	0	0	0
0.180	$3.932\times 10^{-6}$	0.063	$5.578\times 10^{-6}$	0.52	$2.092\times 10^{-6}$	0.054	6.144E−07
0.324	$7.410\times 10^{-6}$	0.140	$1.051\times 10^{-5}$	0.101	$3.942\times 10^{-6}$	0.098	$1.158\times 10^{-6}$
0.537	$1.175\times 10^{-5}$	0.200	$1.667\times 10^{-5}$	0.126	$6.251\times 10^{-6}$	0.171	$1.836\times 10^{-6}$
0.728	$1.608\times 10^{-5}$	0.285	$2.281\times 10^{-5}$	0.194	$8.554\times 10^{-6}$	0.216	$2.512\times 10^{-6}$

**Table 2:** Boundary conditions for the bar.

Prescribed Displacements (mm)			
**Support**	$u_{x}$	$u_{y}$	$u_{z}$
A	$8.67\times 10^{-2}$	1.49	0.00
B	unrestrained	1.22	unrestrained
C	unrestrained	0.51	unrestrained

**Table 3:** Strain component $\epsilon_{xx}$.

Position	Optical Fiber Sensor	BE Analysis I	BE Analysis II
**S021**	$-5.0690000\times 10^{-6}$	$-4.8500933\times 10^{-6}$	$-4.8451565\times 10^{-6}$
**S022**	$-7.2030000\times 10^{-6}$	$-7.4465427\times 10^{-6}$	$-7.4515548\times 10^{-6}$
**S023**	$-2.7100000\times 10^{-6}$	$-2.4041269\times 10^{-6}$	$-2.7457345\times 10^{-6}$
**S024**	$-7.9310000\times 10^{-6}$	$-7.0391310\times 10^{-6}$	$-6.9710208\times 10^{-6}$
